# Targeting KRAS: The Elephant in the Room of Epithelial Cancers

**DOI:** 10.3389/fonc.2021.638360

**Published:** 2021-03-11

**Authors:** Valeria Merz, Marina Gaule, Camilla Zecchetto, Alessandro Cavaliere, Simona Casalino, Camilla Pesoni, Serena Contarelli, Fabio Sabbadini, Monica Bertolini, Domenico Mangiameli, Michele Milella, Vita Fedele, Davide Melisi

**Affiliations:** ^1^ Digestive Molecular Clinical Oncology Research Unit, University of Verona, Verona, Italy; ^2^ Medical Oncology Unit, Santa Chiara Hospital, Trento, Italy; ^3^ Section of Medical Oncology, Università degli Studi di Verona, Verona, Italy

**Keywords:** KRAS, NSCLC, pancreatic cancer, colon cancer, G12C mutation

## Abstract

Mutations of the proto-oncogene KRAS are the most frequent gain-of-function alterations found in cancer. KRAS is mutated in about 30% of all human tumors, but it could reach more than 90% in certain cancer types such as pancreatic adenocarcinoma. Although historically considered to be undruggable, a particular KRAS mutation, the G12C variant, has recently emerged as an actionable alteration especially in non-small cell lung cancer (NSCLC). KRAS^G12C^ and pan-KRAS inhibitors are being tested in clinical trials and have recently shown promising activity. Due to the difficulties in direct targeting of KRAS, other approaches are being explored. The inhibition of target upstream activators or downstream effectors of KRAS pathway has shown to be moderately effective given the evidence of emerging mechanisms of resistance. Various synthetic lethal partners of KRAS have recently being identified and the inhibition of some of those might prove to be successful in the future. The study of escape mechanisms to KRAS inhibition could support the utility of combination strategies in overcoming intrinsic and adaptive resistance and enhancing clinical benefit of KRAS^G12C^ inhibitors. Considering the role of the microenvironment in influencing tumor initiation and promotion, the immune tumor niche of KRAS mutant tumors has been deeply explored and characterized for its unique immunosuppressive skewing. However, a number of aspects remains to be fully understood, and modulating this tumor niche might revert the immunoresistance of KRAS mutant tumors. Synergistic associations of KRAS^G12C^ and immune checkpoint inhibitors are being tested.

## Introduction

Using an oversimplified description, cancer could be defined as a disease caused by the accumulation of alterations in genes coding for proteins involved in cell growth induction or control defined oncogenes or tumor suppressor genes, respectively. In the last half-century, the largest efforts made in the field of experimental targeted therapeutics have been mainly focusing towards the development of therapeutic agents capable of inhibiting oncogenes or restoring the function of tumor suppressor genes. The most important successes in cancer treatment have been, indeed, represented by experimental therapeutics able to effectively interfere with the product of some of the most relevant oncogenes in human cancers. In this regard, those human cancers for which the development of appropriate targeted therapeutics has been most frustrating are sustained in their proliferation by altered genes whose function is essential for the integration and the transduction of physiologic signals in normal cells.

*KRAS* has been the first oncogene identified in human cancer in 1982 (1). Mutations affecting members of the RAS family genes (*KRAS*, *HRAS*, *NRAS*) are the most frequent genetic alterations in human cancers accounting for about 27% of all tumors. *KRAS* mutations are involved in the pathogenesis of different epithelial cancer histotypes, including lung and colorectal cancer, but its role has been especially investigated in pancreatic ductal adenocarcinoma, which is considered the type of tumor mostly dependent on KRAS for its development, metastatic progression, and treatment resistance (2–5).

Because of its high incidence in different tumors and its role in cancer initiation and progression, many efforts have been made in finding effective treatments directly or indirectly targeting KRAS. However, due to the lack of accessible binding pockets and its complex downstream signaling, most of the efforts in targeting KRAS have failed, and mutated KRAS still remains an undruggable target.

Here, we describe and discuss the most recent efforts aimed to identify novel therapeutic approaches of mutated KRAS-driven tumors.

## The RAS/MAPK Pathway

*KRAS* gene encodes for a small GTPase that in normal cells functions as a molecular switch between an active and an inactive state. In quiescent cells, KRAS is inactive and GDP-bound, while in cells receiving extracellular stimuli it is active and GTP-bound. KRAS in its active state leads to the activation of a number of different intracellular transduction signaling pathways, including MAPK and AKT pathways. The switching between inactive and active state is mediated by the guanine nucleotide exchange factors (GEFs) which allow GTP loading. Conversely, the inactive state is mediated by GTPase-activating proteins (GAPs) through GTP hydrolysis (6). GTP-bound RAS interacts and recruits RAF, promoting its accumulation at the plasma membrane and inducing its dimerization and activation of RAF kinases. Activated RAF phosphorylates MEK1 and MEK2 kinases, that consequently phosphorylate and activate ERK1 and ERK2 kinases. ERK1/2 translocate into the nucleus where they phosphorylate several transcription factors that regulate the expression of genes involved in proliferation and cancer progression. MAPK pathway is a linear cascade characterized by complex regulatory mechanisms and feedback loops controlling several kinases. The attempt of inhibiting MAPK pathway, in order to block proliferation signaling, generates cross-talk between different pathways and the activation of compensatory pathways such as the PI3K-AKT-mTORC1 signaling. Noteworthy, PI3K-AKT-mTORC1 pathway, unlike MAPK pathway, can also be activated independently from KRAS, by receptor tyrosine kinases (RTKs) or G-protein coupled receptors (GPCR) and integrin signaling (7).

KRAS mutated cancer cells carry mostly missense mutations causing single amino-acid substitutions in three hotspots, glycine12 (G12), glycine13 (G13), and glutamine61 (Q61). These mutations prevent GAPs from accessing GTP so that hydrolysis is blocked, resulting in a persistently activated GTP-bound state. KRAS activity becomes therefore independent from extracellular stimuli, resulting in overstimulation of downstream pathways and induction of signals for cell proliferation, migration, and metastasis (8). Interestingly, different KRAS mutations can reflect differences in signaling and oncogenic mechanisms, that can have a role in tailoring treatments. In an *in vitro* colorectal cancer study, phosphotyrosine proteomic profiles comparison between the two most frequent KRAS mutations, *KRAS* ^G12D^ and *KRAS* ^G13D^, has been performed. *KRAS* ^G12D^ mutation enhances membrane and adherens junction signaling, while *KRAS* ^G13D^activates signaling molecules such as MAPK kinases, non-receptor tyrosine kinases, and regulators of metabolic processes (9).

## Direct Targeting of KRAS

*KRAS* mutations types and incidence vary among epithelial cancer histotypes. Whereas *KRAS* ^G12C^ mutations are frequent in lung adenocarcinoma, they are rare in pancreatic ductal adenocarcinoma (PDAC). PDAC are enriched in *KRAS* ^G12D^, *KRAS* ^G12V^, and *KRAS* ^G12R^ point mutations (10). These differences are crucial for the development of new potential therapeutic strategies.

Recently, encouraging results using direct KRAS^G12C^ inhibitors have been reported. *KRAS* mutation G12C is present in about 13% of lung cancer, 3% of colorectal cancer, and in a smaller percentage of other epithelial tumors (11). The mutant cysteine-12 is located next to a cryptic pocket (SWII) in GDP-KRAS. The proximity of this cryptic pocket (SWII) to cysteine-12 has driven the development of covalent inhibitors targeting SWII, getting an allosteric inhibition of cysteine-12. ARS-1620 was the first covalent inhibitor binding SWII pocket of KRAS^G12C-^GDP complex developed (12). Starting from this pioneering milestone, many efforts have been made in order to improve potency, drug permeability, solubility, and oral bioavailability and create suitable drugs for clinical use. As a result of these efforts several drugs have been developed and tested in preclinical and clinical studies, including AMG 510 (or sotorasib) and MRTX849 (or adagrasib). Sotorasib was developed by Amgen. Improvements in drug potency have been achieved by taking advantage of an alternative orientation of His95, located in the switch II pocket. The alternative orientation creates a larger surface groove which guarantees an irreversible interaction between KRAS^G12C^ and its inhibitor. The almost complete inhibition of pERK observed upon sotorasib treatment confirmed its enhanced potency (13). Interestingly, while durable responses were obtained in immune-competent tumor-bearing murine models, this activity was not durable in immune-deficient models. Further studies demonstrated that sotorasib induces a pro-inflammatory microenvironment through the expression of chemokines, such as CXCL10 and CXCL11. These chemokines attract tumor-suppressive immune cells, including T cells, macrophages, and dendritic cells, leading to long-term anti-tumor T cells responses. This observation suggested that a more significant and prolonged tumor response could be induced by a combination therapy with immune checkpoint inhibitors (14).

The small molecule covalent inhibitor sotorasib is currently under active clinical development for the treatment of KRAS^G12C^ mutated tumors. Successful results have been recently reported in the phase 1 CodeBreaK100 trial, that investigated sotorasib in patients with advanced and pretreated solid tumors, mainly NSCLC and colorectal cancer, harboring a KRAS^G12C^ mutation (15). About one third of NSCLC patients responded to therapy. Disease control rate was remarkable in both NSCLC (88.1%) and colorectal cancer (73.8%). Median progression free survival was 6.3 months for NSCLC patients and 4.0 months for colorectal cancer patients. Some patients exhibited a rapid disease progression after an initial response, but a group of patients presented durable responses. The lower response rate in colorectal cancer patients (7.1%) suggests a different KRAS dependency across diverse tumor types harboring the same mutation. Sotorasib showed a good safety profile, with diarrhea, fatigue, and nausea as the most common adverse events, and no dose-limiting toxic effects have been observed. Combination treatments of sotorasib with immunotherapy are currently under clinical evaluation and invested of great expectations (NCT04303780, NCT04185883).

A different KRAS^G12C^ covalent inhibitor, named adagrasib, has been developed by Mirati Therapeutics Inc. Adagrasib also binds SWII pocket of GDP-KRAS, inhibiting KRAS pathway and inducing in turn a potent anti-tumor response, as demonstrated in different *in vivo* models. Nonetheless, resistance mechanisms emerged early through the activation of other pathways and activation of compensatory mechanisms, leading to transient or submaximal response to adagrasib. Indeed, high expression or activated mutations of RTKs can activate feedback mechanisms reactivating RAS and stimulating mTOR pathway. Similarly, the co-occurrence of alteration in genes involved in cell cycle regulation, such as CDKN2A and CDK4/6, can induce Rb phosphorylation and cell cycle transition. Based on these evidences, the combination of adagrasib with different drugs, such as EGFR, SHP2, and mTORC inhibitors have been tested in different *in vivo* murine models. The combination of adagrasib with afatinib, RMC-4550, and vistusertib respectively, obtained a stronger inhibition of ERK and S6 phosphorylation than did any single agent treatment with an improved anti-tumor activity. Moreover, the combination of adagrasib and palbociclib decreased Rb and E2F family target genes expression levels, reduced S6 phosphorylation level, and induced major tumor regression, especially in CDKN2A altered models (16). In the phase 1/2 multi-expansion cohort KRYSTAL-1 trial (NCT03785249), adagrasib has been evaluated in patients with advanced solid tumors harboring KRAS^G12C^ mutations, demonstrating an acceptable safety profile and promising clinical activity. In NSCLC patients previously treated with chemotherapy and anti-PD-1/PD-L1 therapy the disease control rate was 96% and objective response rate (ORR) was 45% (https://cm.eortc.org/cmPortal/Searchable/ENA2020/config/normal#!abstractdetails/0000902150). The only commonly reported (>2%) grade 3/4 adverse event was hyponatremia. Disease control was observed in 94% of colorectal patients. Confirmed partial responses (PRs) were observed in a patient with endometrial cancer and a patient with pancreatic cancer (https://cm.eortc.org/cmPortal/Searchable/ENA2020/config/normal#!abstractdetails/0000902140). The most commonly reported adverse events included diarrhea, nausea, fatigue, and vomiting. Other trials have been designed to evaluate the combination of adagrasib with other drugs, such as EGFR, SHP2, or PD1 inhibitors (KRISTAL-1, -2, -7). Among clinical trials conducted in patients with cancers harboring KRAS^G12C^, ARS-3248/JNJ-74699157, LY3499446, and GDC-6036 are being investigated (NCT03114319, NCT04165031, NCT04449874).

PanKRAS inhibitors represent a different category of drugs that do not target a single KRAS isoform selectively but aim to inhibit a broader spectrum of targets. Among these molecules, we count BI 2852, which binds between switch I and switch II pocket and inhibits KRAS interactions with GEFs, GAPs, and its downstream effectors. BI 2852, indeed, effectively reduces pERK and pAKT levels, achieving antiproliferative effects on KRAS mutant cell lines. Because of the high conservation of the SI/II-pocket across RAS isoforms, this molecule can bind with similar affinity most of them (17). SOS1 inhibitors are also panKRAS inhibitors. These drugs, indeed, do not bind directly to KRAS but inhibit the interaction between KRAS: SOS1, preventing KRAS GTP loading and its switching in to the active state. The main representatives are BI 3406 and BI 1701963. BI 3406 has been proven to be active in *in vitro* and *in vivo* murine models, harboring KRAS G12 and G13 codon mutations, but not G12R mutation. SOS1 can be downregulated by ERK-mediated phosphorylation, representing an important negative feedback modulator of KRAS pathway. During treatment with MEK inhibitors, pERK levels reduction induces a decrease of SOS1 phosphorylation, resulting in RAS pathway activation. These observations suggest that inhibition at both levels represents a good strategy to efficiently block KRAS pathway and prevent escape. The combination treatment of SOS1 with MEK inhibitor achieved good results *in vitro* and *in vivo* murine models, with robust pathway inhibition and tumor regression (18). Based on these preliminary preclinical results, BI 1701963, the second representative of SOS1 inhibitors, is being tested, alone and in combination with MEK inhibitor trametinib, in a phase I clinical trial in cancer patients carrying pan-KRAS mutations (NCT04111458).

RAS direct targeting has also been investigated in several studies. The bacterial Ras/Rap1-specific endopeptidase (RRSP) represents a good candidate for RAS direct targeting therapy. RRSP induces a proteolytic cleavage of RAS proteins between residues Tyr-32- and Asp-33 in SWI pocket, this cleavage alters RAS SWI structure, blocking the interaction of RAS with GEFs and preventing the transition of RAS into the active state. It also prevents the interaction with RAF and the downstream signaling pathway (19). RRSP can disrupt both wild type and mutant RAS proteins (including KRAS, HRAS, and NRAS). This blockage allows the inhibition of signal transduction depending from various RAS mutations, overexpression of upstream receptor tyrosine kinases (RTKs), or amplification of wild-type RAS as it happens in head and neck squamous cell carcinoma, esophageal and gastric carcinoma, ovarian adenocarcinoma, and triple-negative breast cancer. To enable the migration of RRSP through the biological membrane, a chimeric toxin formed by RAS/Rap1 specific endopeptidase and the translocation machinery of diphtheria toxin has been developed. The use of this engineered chimeric toxin has achieved good results *in vitro* and *in vivo* murine models, especially in lung and colorectal tumor cell lines and in cells expressing high levels of HB EGF. In human cells HB EGF is highly represented and this could represent a limit for its clinical application, influencing the dose limiting toxicities (DLT). Further engineering steps could allow to overcome this possible limit, aiming to vehicle RRSP across tumoral membrane cells only and sparing normal human cells.

## Indirect Targeting of KRAS Signaling

**Figure 1:**
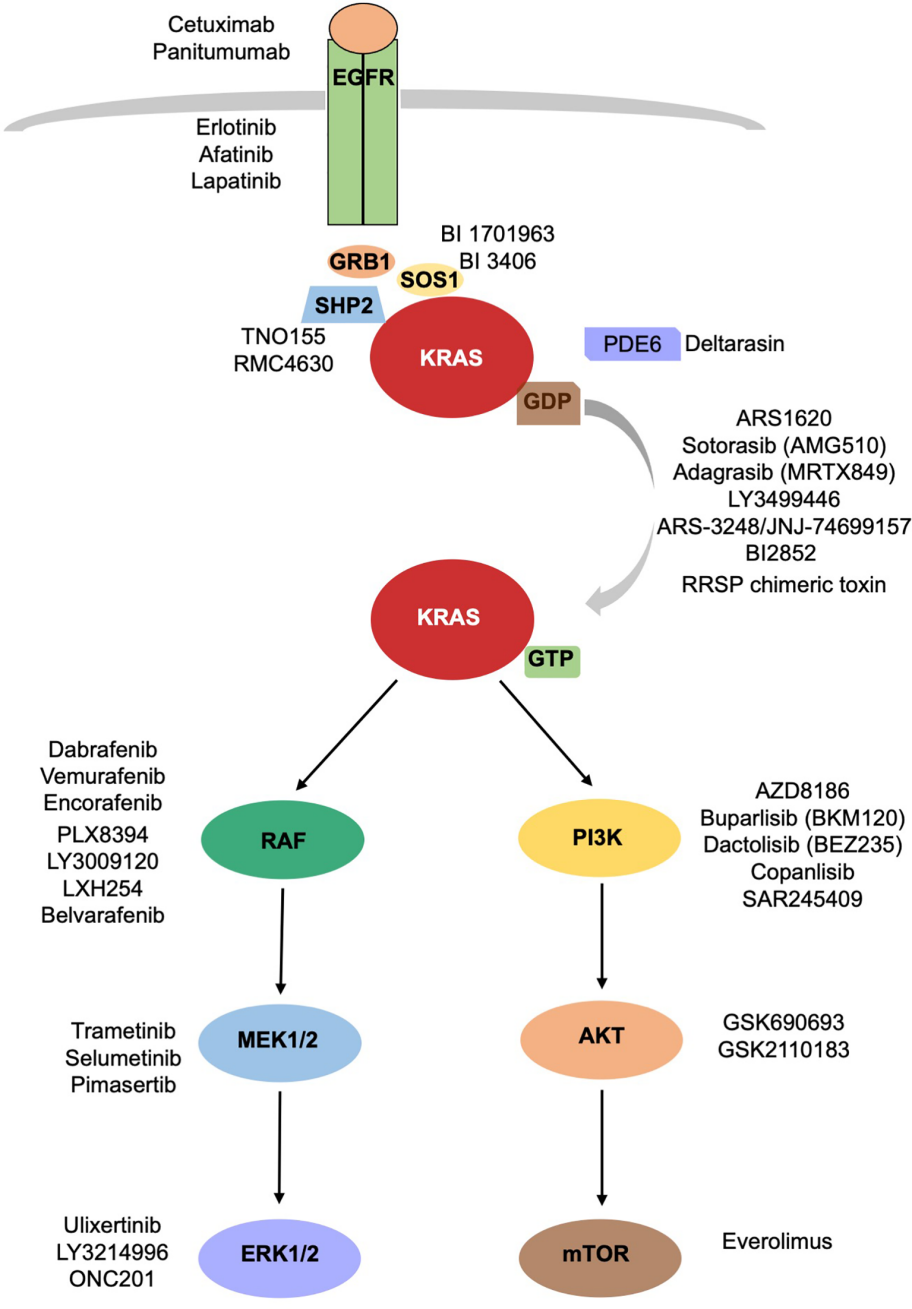
KRAS signaling cascade. Inhibitors of KRAS and upstream and downstream mediators of KRAS are reported.

In the attempt of inhibiting KRAS signaling, different strategies intended to target upstream activators or downstream effectors of KRAS pathway have been developed (**Figure 1**). Because of the redundancy of the intracellular networks involved, the initial enthusiasm for the development of a single target therapy has been mitigated by evidence of emerging mechanisms of resistance. The combination of different drugs targeting different signal pathways could prevent or delay the development of resistance mechanisms, often, however, at cost of increased toxicities.

### Inhibiting KRAS Processing and Activation

Different critical steps are necessary for KRAS activation: nucleotide exchange, localization, processing, effector binding. The blockage of each of them could prevent KRAS activation.

- Nucleotide exchange: The previously described pan-KRAS inhibitors should belong to this category despite their name, since their function is linked to SOS1 (GEF) binding and not KRAS directly. Nucleotide exchange is also favored by SHP2, across their binding to GRB2 and SOS1. Different SHP2 inhibitors, including TNO155 and RMC-4630, directed *versus* non-receptor protein tyrosine phosphatase, are being clinically evaluated in combination therapies in advanced solid tumors (20, 21).

- Processing: prenylation–proteolysis–methylation.

Many efforts have been made to inhibit RAS farnesylation, aiming to prevent its membrane localization. These efforts led to the development of tipifarnib, a small molecule farnesyl transferase inhibitor (22). However, tipifarnib has been evaluated in HRAS mutated cancers only, since KRAS is prenylated by geranylgeranyl transferase and does not need farnesylation for membrane localization. Unfortunately, a dual inhibition of both transferases did not inhibit KRAS prenylation in human patients (23).

- Localization: KRAS splices into KRAS 4A and KRAS 4B. KRAS4B needs a chaperone, PDE6d, to translocate to the membrane surface. Deltarasin, a PDE6d inhibitor, prevents PDE6d from binding to KRAS, causing accumulation of KRAS 4B onto endomembranes (24). It is not clear yet if the cellular effects are due to KRAS inhibition or to other PDE6d effectors inhibition.

### Targeting Downstream Mediators of Intracellular Signaling

Activated KRAS induces RAF proteins phosphorylation and dimerization with consequent activation of their kinases. There are three isoforms of RAF, represented by BRAF, RAF1/CRAF, and ARAF. Numerous studies conducted on BRAF, which is the best characterized isoform, have led to the development of therapies targeting V600E mutation specifically. These targeted therapies are widely used in melanoma and recently have been approved for the treatment of BRAF mutated NSCLC. Unfortunately, the application of BRAF inhibitors vemurafenib, dabrafenib, and encorafenib has failed in KRAS driven tumors, because of the paradoxical activation of ERK1/2. BRAF inhibitors, indeed, bind to BRAF and induce the heterodimerization BRAF/RAF1. The binding of BRAF inhibitors to BRAF mediates an allosteric activation of RAF1, with consequent MEK/ERK activation (25). Novel panRAF inhibitors, known as paradox breakers, have been developed to overcome this effect. Among these panRAF inhibitors we count PLX8394, which seems to have higher affinity with BRAF homodimers and BRAF/RAF1 heterodimers (26, 27), and LY3009120, which blocks the kinase activity of RAF dimers (28). Despite the promising data seen in preclinical studies (29, 30), LY3009120 failed to demonstrate efficacy in early clinical trials as monotherapy. A phase I study conducted in patients affected by RAS or BRAF mutated advanced tumors reported as best response stable disease in 8 of 51 patients (15%) with no complete or partial response achieved (31). LXH254 and belvarafenib are panRAF inhibitors also in active clinical development. A phase I clinical trial with LXH254 alone and in combination with an anti PD1 antibody is ongoing (NCT02607813). Belvarafenib has been tested in a phase I study including patients affected by advanced solid tumors harboring RAS or BRAF mutations, demonstrating good safety profile and antitumor activity (32). A study exploring its use in combination with anti-MEK agents is ongoing (NCT03284502).

MEK inhibitors, such as selumetinib, have been tested in KRAS mutated NSCLC as single agents and in combination with chemotherapy, without showing any clinical benefit (33). The same results have been seen with trametinib (34) and pimasertinib (35) in pancreatic cancer. This failure has been attributed partially to vertical compensation mechanisms of upstream elements, such as RTKs, BRAF, or KRAS, that lead to ERK reactivation, or alternative compensatory mechanisms as the activation of PI3K-AKT-mTORC1 pathway.

ERK inhibitors, such as ulixertinib/BVD523 and LY3214996, have been tested in phase I clinical trials (NCT02608229, NCT02857270). Ulixertinib has recently moved to phase II clinical development. The frequent reactivation of ERK observed during treatment with MEK or BRAF inhibitors calls for a better characterization of ERK inhibitors. This class of inhibitors is being studied accurately, to evaluate their employment in vertical combination (2, 36). Other strategies have been tested to target simultaneously different molecules aiming to vertical combination (NCT01229150, NCT02230553, NCT02258607, NCT04185883, NCT04330664) (**Table 1**).

**Table 1:** Clinical trials investigating combination treatments reported as “Drug 1” and “Drug 2” that target effectors of the same pathway (vertical combination). For each trial the study phase and the setting of patients is indicated.

Clinical trial	Phase	Drug 1	Drug 2	Indication
NCT04185883 (CodeBreak101)	1	AMG510 or sotorasib (KRAS-^G12C^ inhibitor)	PD1 inhibitor PD1 inhibitor MEK inhibitor SHP2 inhibitor pan-ErbB inhibitor EGFR inhibitor + chemotherapy EGFR inhibitor + chemotherapy PDL1 inhibitor Chemotherapy MEK + EGFR inhibitor mTOR inhibitor CDK inhibitor	Solid tumors NSCLC Solid tumors Solid tumors NSCLC Solid tumors Colorectal cancer NSCLC NSCLC Colorectal cancer Solid tumors Solid tumors
NCT04330664 (KRISTAL-2)	1/2	MRTX849 or adagrasib (KRAS^G12C^ inhibitor)	TNO155 (SHP2 inhibitor)	Solid tumors
NCT03785249 (KRISTAL-1)	1/2	MRTX849 or adagrasib (KRAS^G12C^ inhibitor)	Pembrolizumab Cetuximab Afatinib	NSCLC Colorectal cancer NSCLC
NCT04613596 (KRISTAL-7)	2	MTRX849 or adagrasib (KRAS^G12C^ inhibitor)	Pembrolizumab	NSCLC
NCT04111458	1	BI 1701963 (pan-RAS inhibitor)	Trametinib (MEK inhibitor)	Solid tumors
NCT01229150	2	AZD6244 (MEK inhibitor)	Erlotinib (EGFR inhibitor)	NSCLC
NCT02230553	1/2	Trametinib (MEK inhibitor)	Lapatinib (ErbB1-2 inhibitor)	NSCLC
NCT02857270	1	LY3214996 (ERK inhibitor)	Encorafenib + Cetuximab Abemaciclib Chemotherapy	Solid tumors
NCT03284502	1	HM95573 or belvarafenib (RAF inhibitor)	Cobimetinib (MEK inhibitor)	Solid tumors

The other pathway activated by KRAS is the PI3K-AKT-mTORC1 pathway, important for cellular proliferation, motility, and survival. Its persistent activation due to KRAS mutation contributes to cancer progression. Class I PI3K phosphorylates PIP2, which attracts AKT to plasma membrane and induces mTOR activation. Inhibition of PI3K pathway using AKT inhibitors failed in *in vitro* and *in vivo* trials, perhaps because of the activation of other signaling pathways. The compensatory mechanisms between the two pathways, MAPK and PI3K pathway, that emerge blocking one of them, led to the idea of blocking both pathways simultaneously. The combination of MEK inhibitors and AKT inhibitors showed promising results in pancreatic cancer in *in vitro* and *in vivo* studies (37). In a similar way, the combination of PI3K inhibitors with ERK inhibitors (38), MEK inhibitors (NCT01363232, NCT01337765, NCT01392521, NCT01390818) or RAF inhibitors are being tested with promising results in early clinical trials. However, at cost of higher toxicity associated to the double treatment (39).

## Synthetic Lethal Partners of KRAS

Since direct inhibition of KRAS has been proven to be exceptionally challenging, one potential strategy to target tumors dependent upon this oncogene has been through the identification of its synthetic lethal partners. Synthetic lethal partners are genes that if mutated individually are compatible with viability, but the simultaneous perturbation of their expression or pharmacological inhibition of their products determines cell death (40). Synthetic lethality can be exploited in order to target tumor cells harboring undruggable mutations. An example of synthetic lethality is represented by the sensitivity of BRCA mutant cells to PARP inhibition (41). Synthetic lethal partners of KRAS could be downstream of its pathway or acting in parallel adaptative signaling. Targeting synthetic lethal partners should reduce the risk of adverse events because mutated cancer cells are more sensitive to this strategy compared to normal cells (42).

Among the different methods used to identify novel KRAS synthetic lethal partners, RNA interference has been the approach initially and more frequently applied (42). Unfortunately, evidence suggests that the reproducibility of this technology could be limited by the library quality and off target effects (43). More recently, CRISPR-Cas9 screening technology has been applied to loss-of-function genetic screening, enabling the complete knockout of target genes, that has been useful in identifying essential genes in KRAS mutant cancer cells (44). Results from different studies looking for synthetic lethal partners are scarcely overlapped and attempts to reproduce published KRAS synthetic lethal targets failed (45, 46). The novel CRISPR-Cas9 technology improves genetic perturbation but is not able alone to overcome limitations associated with different cellular and genetic contexts. Interestingly, a meta-analysis of published synthetic lethal screens highlighted that different studies’ results overlap at the pathway rather than at a gene level (47). Furthermore, these differences have been ascribed to changes in genetic context or cellular conditions.

Several studies have identified different putative KRAS synthetic lethal partners (**Table 2**). Inhibitors of the MAPK pathway showed greater sensitivity in KRAS mutated cancers compared to the wild-type ones (58). Probably because of their sole cytostatic effect, MEK inhibitors have proved no clinical efficacy as single agents in RAS mutant cancers (59). The combination of a MEK inhibitor with an inhibitor of the antiapoptotic BCL-XL led to increased apoptosis in many KRAS mutant cell lines from different histologies, and tumor regression in *in vivo* lung cancer mouse models (48). A phase 1b/2 trial is investigating the safety, pharmacokinetics, pharmacodynamics, and clinical activity of the combination of the MEK inhibitor trametinib and the BCL2-family inhibitor navitoclax (ABT-263) in patients with KRAS or NRAS mutated advanced solid tumors (NCT02079740). An interim analysis showed a good safety profile and initial signs of efficacy, in particular in gynecologic tumors. Evidence coming from other *in vitro* and *in vivo* studies demonstrated efficient induction of apoptosis in KRAS or BRAF mutant colorectal cancer cell lines treated with navitoclax in combination with the TORC1/2 inhibitor AZD8055 but not in the wild-type controls. Similar results were obtained in murine models (60). IGF1R and MEK inhibition resulted in growth inhibition of KRAS muted NSCLC cell lines and murine tumors (61). FGFR1 inhibition combined with the MEK inhibitor trametinib has shown to mediate cell death in KRAS-mutant lung cancer both *in vitro* and *in vivo* (49).

**Table 2:** RAS synthetic lethal partners and KRAS mutant cell lines in which they have been identified. Synthetic lethal gene inhibition is reported if tested.

Synthetic lethal genes or pathways	Cell lines	Drug inhibition
BCL-XL (BCL2L1) (48)	KRAS mutant cell lines from different histologies	MEK inhibitor plus BCL-XL inhibition
FGFR1 (49)	KRAS mutant lung cancer	MEK inhibitor plus FGFR1 inhibition
CDK4 (50)	KRAS mutant NSCLC	Not tested*
AKT (51)	KRAS mutant pancreatic cancer	AKT and glutathione synthesis inhibition
TBK1 (52)	KRAS mutant NSCLC	TBK1 inhibition
XPO1 (53)	KRAS mutant NSCLC	XPO1 inhibition
TAK1 (54)	KRAS mutant colon cancer	5Z-7-oxozeaenol
YAP1 (55)	KRAS pancreatic cancer	Not tested*
WT1 (55)	KRAS mutant lung cancer	Not tested*
GATA2 (56)	KRAS mutant NSCLC	Not tested*
SNAI2 (57)	KRAS mutant colon cancer	Not tested*

*Drug inhibition of the correspondent synthetic lethal gene has not been tested in in vitro or in vivo studies.

CDK4 has been proposed as synthetic lethal partner in KRAS mutant NSCLC. This synthetic lethal interaction was observed only in lung cancer, not in colon or pancreatic cancer, pointing to a different tissue specific dependency of KRAS signaling (50). Targeting AKT and the glutathione antioxidant pathway mimicking Nrf2 ablation inhibited pancreatic adenocarcinoma tumors *ex vivo* and *in vivo* (51). NF-κB pathway has a central role in KRAS mutated cancers (62, 63). RAL-GEF family is one of the effectors of KRAS and mediates the activation of NF-κB, contributing to oncogenesis (64). NF-κB inhibition in a mouse model of lung adenocarcinoma expressing KRAS^G12D^ and lacking p53 has been demonstrated to reduce tumor development (65).

Another putative synthetic lethal partner of oncogenic KRAS is represented by the IκB kinase (IKK)–related kinase Tank-binding kinase-1 (TBK1). TBK1 is activated by RalB, a small GTPase downstream of KRAS belonging to the Ral signaling pathway, and Sec5, a component of the exocyst (66). TBK1 regulates an autocrine CCL5 and IL-6 signaling, inducing carcinogenesis in KRAS mutated cancer (52). Furthermore, activated TBK1 promotes NF-κB signaling through BCL-XL and the c-Rel protooncogene. Inhibiting TBK1 induces cell death in KRAS driven NSCLC adenocarcinoma murine models.

The JAK-STAT signaling pathway has a recognized role in pancreatic cancer development. In KRAS driven pancreatic cancer models, inhibiting JAK1/2 and TBK1 with momelotinib showed preclinical efficacy *in vitro* and *in vivo* (52). Nevertheless, to date it has not exhibited signs of activity in human pancreatic cancer (67).

XPO1 has also been proposed as synthetic lethal partner of KRAS. XPO1, overexpressed in many types of human cancers, is an export receptor in charge of the nuclear-cytoplasmic transport of many proteins. XPO1 has been proposed as a therapeutic target in several tumors including KRAS-mutant lung cancer. The effect of XPO1 inhibition consists in the accumulation of nuclear IκBα and consequent suppression of NFκB activity. Studies conducted on KRAS-mutant NSCLC cells showed that inhibition of the nuclear export XPO1 leads to a synthetic lethal interaction with oncogenic KRAS (53).

TAK1 has been suggested as mutant KRAS synthetic lethal target in colon cancer (54). In APC/KRAS mutant cells, KRAS mediates TAK1 activation and enhances Wnt activity by stimulating BMP-7 secretion and BMP signaling. TAK1 inhibition prompted apoptosis in KRAS-dependent colon cancer cells. However, TAK1 dependency may not be restricted to colon cancer, and approaches in targeting TAK1 have shown activity in other KRAS dependent tumors as well (68–70).

The transcription factor YAP1 is sustained by TAK1 and mediates KRAS independent growth (71, 72). YAP1 has been shown to overcome KRAS blockade to prompt pancreatic cancer growth in murine models (55). Representing a central hub in resistance to RAF and MEK inhibition, targeting YAP1 could represent a combination therapy in KRAS mutated cancers (73).

Loss of the transcription factor Wilms tumor 1 (WT1) has been correlated with decreased proliferation and increased cell senescence in KRAS driven cancer cell lines (55). However, WT1 remains not druggable to date (74).

The transcription factor GATA2 has been identified as a synthetic lethal target in RAS pathway mutant NSCLC models. However, GATA2 itself remains undruggable (56).

Deficiency of the DNA repair machinery has been described in KRAS mutant cells (47). BRCA1 is a strong synthetic lethal partner of PARP inhibition. PARP inhibition has also been proposed as a putative effective strategy in KRAS mutant cells.

RAS signaling is a known mediator of epithelial-mesenchymal transition (EMT). Thus, EMT regulators could represent therapeutic targets in KRAS driven tumors (75). The SNAI2 gene encoding SNAIL, a transcription factor and regulator of EMT, has been identified as a KRAS synthetic lethal target in colorectal cancer cell lines (57).

Direct targeting of KRAS has been approached and the need for targeting synthetic lethal partners could be questioned. However, synthetic lethal partner inhibitors could be used in the future in combination with direct inhibition in order to overcome possible escape mechanisms.

## Targeting Metabolic Reprogramming in KRAS Mutant Cancers

Studies conducted using murine pancreatic cancer models have shown that KRAS^G12D^ stimulates the expression of glucose transporter 1 (GLUT1) and glycolytic enzymes and conveys glucose intermediates into the hexosamine biosynthesis pathway (HBP) and non-oxidative pentose phosphate pathway (PPP). The inhibition of the HBP gene (Gfpt1) or non-oxidative PPP genes (Rpia or Rpe) suppresses the KRAS dependent tumor growth (76). It has been shown that an increase in glucose uptake through enhanced GLUT1 expression is dependent on KRAS and BRAF mutation in colorectal cancer cell lines and sustained their survival (77). Furthermore, glucose deprivation with a glycolysis inhibitor suppressed tumor growth. Mutated KRAS determines higher ^18^F-fluorodeoxyglucose accumulation possibly by upregulation of GLUT1 (78). A retrospective study reported a significantly higher ^18^F-fluorodeoxyglucose accumulation detected with positron emission tomography in KRAS mutant colorectal cancer patients compared with wild-type ones (79). High levels of vitamin C have been found to selectively kill colorectal cancer cells harboring KRAS or BRAF mutations. The increased uptake of the oxidized form of vitamin C through GLUT1 causes oxidative stress and cell death only in KRAS or BRAF mutant cells (80).

Cancer cells are characterized by increased anabolic metabolism, which requires the use of the amino acid glutamine. It has been demonstrated that oncogenic KRAS mediates the reprogramming of glutamine metabolism in pancreatic adenocarcinoma cells by modifying the transcription of metabolic enzymes in a noncanonical pathway of glutamine (81). However, the tissue of origin and the microenvironment can impact on metabolic features. For example, pancreatic cells do not depend on the branched-chain amino acid (BCAA) processing enzymes Bcat1 and Bcat2, which enables BCAAs to be utilized as a nitrogen source, contrary to NSCLC (82). In KRAS mutated colorectal cancer cells the pentose phosphate pathway has been demonstrated to be essential for the growth in aerobic conditions and glutamine conversion into α-ketoglutarate and alanine aminotransferase for KRAS induced anchorage-independent growth (83). In KRAS driven lung cancer mouse models mitochondrial metabolism and mitochondrial reactive oxygen species generation, which is allowed by glutamine conversion into α-ketoglutarate, are essential for KRAS induced tumorigenicity (83). Models obtaining the suppression of KRAS led to reveal potential KRAS independent escape mechanisms. In KRAS G12D mouse model of pancreatic cancer surviving cells responsible for tumor relapse rely on oxidative phosphorylation, making the combined inhibition of the KRAS pathway and mitochondrial respiration a possible therapeutic strategy (84).

Autophagy is a mechanism characterized by degradation of intracellular components. It is stimulated by oxidative stress, nutrient shortage, and protein damage through inhibition of the AMPK and mTOR pathways and the activation of the unfolded protein response system (85). Pancreatic adenocarcinoma tumors show raised autophagy, whose inhibition demonstrated to reduce tumor growth (86). However, the role of KRAS in autophagy remains controversial. In a study conducted in different cancer cell lines, KRAS mutation was not correlated with the dependance to autophagy (87). The use of hydroxychloroquine, that inhibits autophagy preventing lysosome acidification, failed to show therapeutic activity in pancreatic cancer patients (88). However, several studies are ongoing to investigate hydroxychloroquine in combination with chemotherapy in pancreatic cancer (NCT04524702, NCT04132505). The deficiency of atg7, an essential autophagy gene, in KRAS^G12D^ mutated NSCLC mouse models determined the accumulation of dysfunctional mitochondria and inhibited cancer growth (89).

RAS proteins have been demonstrated to enhance macropinocytosis, a process by which extracellular fluid and extracellular proteins are internalized through vesicles. Macropinocytosis inhibition with amiloride blocked the growth of KRAS mutated pancreatic cancer xenografts (90).

The metabolism of fatty acids has been correlated with KRAS mutation in NSCLC. It has been shown that KRAS regulates lipid homeostasis and Acyl-coenzyme A synthetase, an enzyme involved in fatty acid metabolism, essential for mutant KRAS lung cancer tumorigenesis *in vivo* (91). Furthermore, KRAS has been reported to promote lipogenesis through the induction of fatty acid synthase in lung cancer (92).

In KRAS/p53 mutant lung cancer mouse models the inhibition of HSP90 combined with rapamycin was shown to promote endoplasmic reticulum stress and mitochondrial damage and tumor regression (93).

## Putative Escape Pathways to KRAS Inhibition

Although a clinically relevant strategy for effectively targeting KRAS in all of its mutated status seems still far to be developed, potential mechanisms of resistance for KRAS inhibition have been already explored in several preclinical models.

KRAS^G12C^ inhibitors bind specifically to inactive GDP-bound form of KRAS. Thus, the potency of KRAS^G12C^ inhibition is reduced by increased RTK activity, that promotes cycling of KRAS^G12C^ to its active GTP-bound form, hindering KRAS^G12C^ drug inhibition (94). Furthermore, the suppression of nucleotide exchange activity downstream of tyrosine kinases enhances KRAS^G12C^ inhibition, suggesting possible combination strategies.

By targeting KRAS^G12C^ with ARS-1620, the phosphorylation of multiple RTKs was augmented in different ways across diverse KRAS^G12C^ mutant models (95). Synergistic effects of RTK inhibitors combined with KRAS blockade may vary across different tumor cell types (96).

KRAS^G12C^ inhibitors induce growth inhibition mainly by targeting MAPK/ERK pathway. The redundancy of parallel growth factor signals can bypass KRAS blockade, underlying intrinsic resistance to KRAS^G12C^ inhibitors (96). However, combining this strategy with the inhibition of SHP2, a phosphatase that mediates signaling of different RTKs to KRAS, blocked the feedback reactivation and enhanced efficacy of KRAS^G12C^ inhibition *in vitro* and *in vivo*, also in models refractory to KRAS^G12C^ inhibition alone (16). This encouraging preclinical evidence led to move to an early-phase clinical trial investigating combination therapies aimed to simultaneously targeting KRAS^G12C^ and SHP2 (NCT04330664, NCT04185883). Another central node stimulated by RTK is represented by SOS1, a guanine nucleotide exchange factor activating KRAS (97). The SOS1 inhibitor BAY-293 can synergize with the KRAS^G12C^ inhibitor ARS-853 reducing cell proliferation (98).

Noteworthy, the scenario of KRAS mutated cancer is extremely heterogeneous and complex. The dependency on KRAS signaling varies across different KRAS mutant cancer types and could reflect the variability in the tumor response, representing a possible mechanism of intrinsic resistance (75, 99). KRAS^G12C^ colorectal cancer cells have been shown to have higher basal EGFR activity compared to NSCLC cells, leading to higher phospho-ERK rebound and thus resistance to KRAS^G12C^ blockade (100). This finding is consistent with clinical results, in which activity of sotorasib seems to be lower in colorectal cancer patients. Thus, combining KRAS^G12C^ inhibition with EGFR inhibition could represent an effective treatment strategy. Indeed, in KRAS mutant cancer cells KRAS^G12C^ inhibition with ARS-853 was increased by the combination with EGFR inhibitors (94).

Other adaptive resistance mechanisms for KRAS^G12C^ inhibition involved reactivation of MAPK pathway and failed PI3K–AKT pathway inactivation (96). The combination of the KRAS^G12C^ inhibitor ARS1620 with PI3K inhibition has demonstrated to be effective *in vitro* and *in vivo* in different models resistant to single-agent KRAS^G12C^ inhibitor. Also a strategy of blocking PI3K effectors, such as AKT and mTOR, together with KRAS^G12C^, proved to be effective in preclinical studies (94, 101).

Activation of RTK signaling in KRAS^G12C^ mutant cancers could limit the KRAS^G12C^ therapeutic inhibition both by increasing regulation of GTPase activity and promoting KRAS independent ERK and mTOR/S6 pathway activation (16). The combination of the mTOR inhibitor vistusertib with the KRAS^G12C^ inhibitor MRTX849 also improved antitumor activity *in vitro*.

mTOR and IGF1R could also play a central role in KRAS inhibition resistance. The addition of mTOR and IGF1R to the KRAS^G12C^ inhibitor ARS1620 improved efficacy in KRAS^G12C^ mutant lung cancer in *in vitro* and *in vivo* mouse models.

Another mechanism proposed for the adaptive resistance to KRAS^G12C^ inhibitors is represented by feedback reactivation of wild-type RAS (95). In KRAS^G12C^ models, an adaptive RAS pathway reactivation after a rapid KRAS^G12C^ inhibition with ARS-1620 and AMG-510 is driven by activation of wild-type RAS (NRAS or HRAS) mediated by RTKs and is not inhibited by KRAS^G12C^ inhibitors.

In response to KRAS^G12C^ inhibitors, proliferation of cancer cells can be resumed through the production of new KRAS^G12C^ (102). The distribution of newly synthetized KRAS^G12C^ between the active and inactive state, which is the only conformation bound by KRAS^G12C^ inhibitors, modulated the divergent response. Cells producing new KRAS^G12C^, which is converted to the active and drug insensitive state, are able to escape KRAS^G12C^ inhibition.

Another possible mechanism responsible for resistance to KRAS^G12C^ inhibitors is represented by the presence of additional KRAS genetic alterations that can potentiate nucleotide exchange or impair inherent GTPase activity (94). Furthermore, the resistance to KRAS^G12C^ inhibitors could be cause by the presence of a heterogeneous spectrum of KRAS mutations in the same patient (103).

Moreover, aurora kinase A (AURKA) was shown to promote drug inhibition escape by interacting with KRAS^G12C^ and c-Raf (102). In KRAS^G12C^ mutant cancer models a synergic effect was demonstrated with the KRAS^G12C^ inhibitor ARS-1620 and the AURKA inhibitor alisertib (102).

In an inducible KRAS^G12D^ pancreatic cancer mouse model, the amplification and overexpression of the transcriptional coactivator Yap1 has been demonstrated to be a potential KRAS independent bypass mechanism (55). In this study, indeed, after KRAS extinction and complete tumor regression in all mice, about two thirds of them relapsed. At least three possible resistance mechanisms have been identified. In about half of the relapsed tumors, a KRAS transgene amplification has been found, meaning that genomic alteration on target itself could bypass target blockade. Another possible mechanism leading to tumor relapse is represented by the compensatory activation of other key growth pathways. According to this, previous findings showed that expression of receptor tyrosine kinases bypasses the KRAS dependency (75). Furthermore, a novel mechanism of resistance to KRAS inhibition through a Yap1-mediated transcriptional program has been proposed. Although Yap1 is not sufficient for driving *de novo* pancreatic cancer development, it can drive tumor recurrence in inducible KRAS^G12D^ pancreatic cancer models (104).

Since increased cell proliferation and antiapoptotic signaling could represent a possible mechanism of resistance to KRAS^G12C^ inhibitors, their combination with chemotherapy, that inhibits cell proliferation, could boost responses and deter resistance. There are also evidences showing a synergistic effect of cell cycle inhibitors like palbociclib in combination with KRASG12C inhibitors (14). Indeed, genetic alterations in CDKN2A, CDK4, or CCND1 can be found in up to 20% of KRAS mutated NSCLC cancers (105).

Overall, these data support the utility of combination therapies in overcoming intrinsic and adaptive resistance and enhancing clinical benefit of KRAS^G12C ^inhibitors.

## KRAS Reprogramming of Tumor Microenvironment and Potential Implication for Immunotherapeutic Approaches

The development and progression of tumors depend not only on oncogenic mutations but also on the interaction with the surrounding microenvironment, which creates a nurturing niche for cancer cells. KRAS mutant tumors are typically characterized by an immunosuppressive state (106). KRAS signaling induces in tumor cells the expression of immunomodulatory factors and inflammatory cytokines, with subsequent recruitment of neutrophils and myeloid-derived suppressor cells (MDSCs), creating an immunosuppressive tumor microenvironment. KRAS^G12D^ was shown to induce ELR CXC chemokines in human embryonic kidney cells (107). Large production of chemokines was observed also in KRAS mutant pancreatic cell lines (108). In murine lung cancer models KRAS^G12D^ demonstrated to stimulate CXCL1, 2, and 5, leading to neutrophils and macrophages infiltration (109). A tumor growth promoting role for CXCL2 and CXCL5 was also found in KRAS mutated pancreatic cancer cell lines (110).

The binding of CXCL3 with CXCR2 and the production of GM-CSF induce the accumulation of MDSCs. In colorectal cancer models KRAS^G12D^ has shown to downregulate the expression of interferon regulatory factor 2 (IRF2), which in turn suppresses CXCL3 expression, resulting in high expression of CXCL3 and promoting migration of myeloid-derived suppressor cells to the tumor microenvironment (111). Responsiveness to anti-PD-1 therapy was increased in colorectal cancers with higher IRF2 expression. The tumor microenvironment is populated by other myeloid cells, such as alternatively activated immune suppressive M2 macrophages, and lymphoid cells, including CD4+FoxP3+ T regulatory (Treg) cells, CD19+IL-10+ B regulatory (Breg) cells, and interleukin (IL)-17-producing T helper (Th)17 cells (112, 113).

IL-6 expression has been correlated with KRAS mutated signaling and seems to play a central role in shaping the immune milieu. In pancreatic cancer models IL-6 signaling was accompanied by an infiltration of myeloid cells and lymphocytes (114).

Upregulation of IL-10 transcription through MEK/ERK/AP-1 pathway was shown in KRAS mutant colorectal cancer cells and its secretion was required for the conversion of CD4+ T cell to CD4+FoxP3+ Treg cells (113). High IL-10 levels were associated with a worse prognosis in patients with KRAS mutated cancers (106).

The capacity of TGF-β in regulating the immune system and inhibiting inflammation is acknowledged since many years (115). Either RAS downstream MAPK and PI3K pathways seem to contribute to TGF-β production (116). In KRAS mutated colorectal cancer lines TGF-β secretion was required for Treg cell differentiation as mediated *via* the MEK/ERK/AP-1 pathway (117). In a lung cancer mouse model, it has been demonstrated that IL-10 and TGFβ secreted by KRAS mutated cancer cells, induce the conversion of CD4+ CD25- T-cells into FOXP3+/CTLA4+/CD122+ T regulatory cells (Tregs) (117). In immune-excluded colorectal cancer models the inhibition of TGF-β promoted anti-tumorigenic immune infiltration, restoring sensitivity to PD-L1/PD-1 blockade (118). Considering that pancreatic cancer is a poorly immunogenic, “cold” tumor, novel approaches targeting the microenvironment have been explored. Signals of activity using TGF-β-inhibitor galunisertib in combination with gemcitabine have been showed in advanced pancreatic cancer patients (119). Moreover, conventional therapy is able to shape the immune landscape in KRAS mutant tumors. It has been demonstrated that mutant KRAS pancreatic cancer cell lines treated with chemotherapy activate MAPK and NF-κB pathways, inducing the secretion of inflammatory cytokines able to enhance monocyte differentiation towards MDSCs and thus counteracting therapy response (120). Other mechanisms have also been proposed. High circulating IL-8 levels have been suggested to be a potential predictive biomarker of resistance to nanoliposomal irinotecan (nal-IRI) in gemcitabine-refractory patients with pancreatic cancer (121). Nal-IRI has been developed to exploit tumor-associated macrophages (TAMs) for accumulation and conversion into its active metabolite. IL-8 has shown an increased mobilization of immature CD11b^+^Gr-1^+^ myeloid cells, thus, it has been hypothesized that high IL-8 levels and low TAMs activity could be correlated with lack of nal-IRI activity (122).

Mutated KRAS has a central role in pancreatic cancer development and growth through regulation of T cell cytokines in the microenvironment, therefore shaping the metabolic cancer cell landscape (123). The presence of T cells in the microenvironment is of crucial importance considering their therapeutic potential with immune checkpoints inhibitors. T_H_1 cells are generally associated with response to immunotherapy and promote CD8+ T cell infiltration (124). T_H_2 cells prevent tumor rejection and promote tumor growth (125). In addition to promoting macrophage M2 polarization, IL-4, which is abundantly produced by T_H_2 cells, has been recently demonstrated to stimulate tumor cell proliferation through KRAS in pancreatic cancer. Mutant KRAS in cancer cells stimulates cytokine receptor expression such as such as IL4R, IL2Rγ, and IL13Rα1 that, in turn, facilitate the Jak1-Stat6-cMyc pathway activation by IL-4 and IL-13. cMyc, which is activated by Stat6, is required for metabolic reprogramming and drives glycolysis.

GM-CSF can exert both immune suppression and stimulation and the balance could be dependent on its levels (106). KRAS^G12D^ is responsible for GM-CSF transcription through MAPK and PI3K pathways in pancreatic cancer cells (126). The correlation between reduced overall survival and high levels of GM-CGF observed in pancreatic cancer patients is probably due to the ability of GM-CSF to cause MDSC differentiation and inhibition of T cell proliferation (120).

Although IL-10 and TGF-β can induce a shifting of macrophages towards the alternative activated immunosuppressive M2 state, a clear correlation between their secretion by KRAS mutated cancer and macrophage polarization has not been established (106). In pancreatic cancer both M1 and M2 macrophage phenotypes have been hypothesized to play an important role in tumor initiation and progression and growth (127). In advanced pancreatic cancer macrophages represent the most abundant immune cell population, playing mainly an immunosuppressive role (128). The correlation of macrophages with prognosis in lung cancer patients remains controversial (129). Mechanisms of macrophage recruitment in KRAS mutant lung cancer are not well defined, but it has been hypothesized a role for CXCR2 signaling (130).

Also, a crosstalk between cancer-associated fibroblasts (CAFs) and KRAS mutant cancer cells has been shown. In a KRAS^G12D^ mutant lung cancer and CT26 colon cancer mouse models, the depletion of fibroblast activation protein (FAP), expressed by CAFs, was demonstrated to inhibit tumor cell proliferation through accumulation of collagen and decrease of myofibroblast content and blood vessel density (131). In pancreatic cancer cells KRAS activates Hedgehog pathway, which is involved in the generation and maintenance of the typical dense tumor stroma (132). In a pancreatic cancer mouse model, mutant KRAS induced the expression of Sonic hedgehog, which in turn activated the transcription factor GLI1. GLI1 regulates IL-6 expression in fibroblasts by binding its promoter and IL-6/STAT3 axis is involved in pancreatic carcinogenesis (133).

Pancreatic stellate cells are essential in disease progression and are the most represented cell type of tumor stroma (134). TGF-β and many other factors secreted by pancreatic cancer cells contribute to the activation of stellate cells which, in turn, produce and release several other growth factors and cytokines (106). Pancreatic stellate cells and mutant KRAS cancer cells have a synergistic effect on the immune microenvironment.

The composition of the immune population and its crosstalk with KRAS altered tumor cells have a central role not only in determining tumor onset and progression but also in sensitivity to immunotherapeutic drugs (135). A study reported that oncogenic RAS signaling can upregulate PD-L1 expression on tumor cells through a mechanism of increased PD-L1 mRNA stability (136). Indeed, KRAS-induced MEK signaling promotes the inhibition of tristetetrapolin, a negative regulator of PD-1 expression. In human lung and colorectal tumors, RAS pathway activation has been correlated with elevated expression of PD-L1. It has been reported that PD-1 and PD-L1 expression is more frequent in KRAS mutated NSCLC (137). Some studies have already shown a clinical relevance of the combination of MEK inhibitors with immunotherapy (138, 139). An ongoing phase 1b/2 trial is testing the activity of the treatment with MEK inhibitor binimetinib in combination with nivolumab or nivolumab plus ipilimumab in pretreated patients with microsatellite stable metastatic colorectal cancer harboring a RAS mutation (NCT03271047).

Differently from other TKIs, novel KRAS^G12C^ inhibitors are specifically selective for the mutation variant of KRAS and should not have any effects on the immune cells directly. Thus, KRAS inhibition in cancer cells can shift the balance from an immunosuppressive state to a microenvironment favoring effective antitumor activity and can sensitize tumors to checkpoint inhibitor therapy.

The predictive role of KRAS status to immune checkpoint inhibitors in NSCLC is controversial. Although KRAS status has never been included as stratification factor in clinical trials with immune checkpoint inhibitors for NSCLC, a subgroup analysis of the CheckMate 057 trial revealed that patients with tumors harboring a KRAS mutation had a greater clinical benefit with nivolumab compared to docetaxel (140). A meta-analysis conducted on five prospective randomized trials has revealed that (141) KRAS mutation is associated with a better outcome in patients treated with PD-1/PD-L1 inhibitors in second-line setting (142). However, the study failed to prove that KRAS status is an independent predictive factor for treatment. The retrospective IMMUNOTARGET registry confirmed a greater benefit from immune checkpoint inhibitors in patients with KRAS mutated NSCLC compared to those with EGFR mutant tumors (143). Another retrospective study found similar activity of immunotherapeutic agents in KRAS mutated compared to KRAS wild-type lung cancer patients (141). The mutation variants KRAS^G12V^, KRAS^G12D^, and KRAS^G13C^ have been associated with higher tumor expression of PD-L1 compared with other variants in NSCLC.

Interestingly, some evidence supports the hypothesis that STK11/LKB1 co-mutation in KRAS mutated NSCLC could represent a negative predictive factor for immunotherapy (144). LKB1 loss is involved in the suppression of stimulator of interferon genes (STING), determining a decreased expression of type I interferon genes and chemokines that facilitate T-cell recruitment (145). STING activation has been associated with response to immunotherapy and is stimulated by chemotherapy (146). A subgroup of STK11 and p53 co-mutated NSCLC is characterized by high STING- and immune-related gene expression. KRAS mutated tumors with co-occurring CDKN2A/B mutations have a scarce immune infiltrate and low PD-L1 expression, resulting in resistance to anti-PD-1 therapies (147). Another group of KRAS mutant NSCLC presents p53 co-mutation and they also have high PD-L1 expression, high T-cell infiltration and, thus, enhanced response to immunotherapy. For the resistance to anti-PD-1 observed in this latter group, a mechanism involving STAT signaling has been proposed (148). In KRAS/p53 mutant murine lung cancer models neurotrophic receptor tyrosine kinase 1 (NTRK1) has been found to be upregulated after treatment with PD-1 inhibitors and to regulate JAK/STAT signaling, promoting PD-L1 expression and CD8+ T cell exhaustion in the microenvironment.

p21-activated kinase 4 (PAK4) is a serine/threonine kinase acting downstream of RAS signaling. PAK4 overexpression has been found in tumor biopsies of anti-PD-1 non-responders and was correlated with low T cell and dendritic cell infiltration across different cancer types, with a strong negative correlation in pancreatic cancer (https://doi.org/10.1038/s43018-019-0003-0). The genetic knockout of PAK4 augmented tumor infiltration by T cells and natural killer cells and pharmacological inhibition of PAK4 synergized with PD-1 blockade immunotherapy in melanoma mouse models, suggesting the possibility of enhancing the efficacy of immunotherapy also in KRAS mutant tumors.

Therefore, combining KRAS inhibition with immune checkpoint blockade has a strong biological rationale and could open the way to therapeutic options, reversing the innately immunoresistant phenotype of some RAS mutant cancers.

A recent study has suggested that the novel KRAS^G12C^ inhibitor sotorasib (AMG 510) can potentiate immune rejection when combined with anti-PD-1 immune checkpoint inhibitor (14). On one side, sotorasib promotes tumor regression by blocking growth and proliferation pathways, on the other side, it induces a change in the expression of immunomodulating factors in cancer cells, such as increased production of T-cell chemoattractants CXCL10 and CXCL11. The combination of sotorasib with anti-PD-1 determined complete regression in nine out of ten CT26 KRAS mutated colon carcinoma mice, which is one of the most immune-responsive mouse tumor models, and induced T cell memory. The immunological memory was demonstrated by the fact that the growth of isogenic KRAS G12D tumors in treated mice was impaired.

The phase 1b trial CodeBreakTM 101 testing the combination of sotorasib with anti-PD-1 is ongoing in patients with a KRAS^G12C^ advanced solid tumors (NCT04185883).

Further investigation about the synergistic association of KRAS^G12C^ and immune checkpoint blockade is warranted. It has to be explored if this combination will be effective only in tumors that are already moderately sensitive to immunotherapy or even in those intrinsically resistant to immune checkpoint inhibition.

## Conclusions

Although KRAS is the most mutated oncogene in human cancer, it has considered to be undruggable because of its structural biology. Recently, exciting data of activity have been reported with KRAS^G12C^ inhibitors in early-phase clinical trials, raising a growing interest for KRAS inhibition, especially in lung cancer.

Different strategies are being explored in order to overcome resistance mechanisms and enhance the efficacy of KRAS inhibition, for example targeting synthetic lethal partners of KRAS. There is a hope that in the next future it will be achievable to block other mutation variants of KRAS other than G12C, making possible to exploit this approach also in other KRAS mutant tumors. Combinations of KRAS inhibitors and immune checkpoint inhibitors are being tested, since they showed a synergistic effect in a preclinical setting. Considering the immunosuppressive microenvironment characterizing KRAS mutant cancers, results from clinical trials utilizing this mechanism are anxiously awaited.

Many improvements have been made in targeting the oncogene KRAS, that was previously thought impossible to block, paving the way for a novel clinical field of research that will probably lead to new horizons in the future clinical practice.

## Author Contributions

VM and DaM designed the review. VM and MG wrote the manuscript. VM, MG, CZ, AC, SCa, CP, SCo, FS, MB, DoM, MM contributed to literature revision. VF and DaM contributed to the final revision. All authors contributed to the article and approved the submitted version.

## Funding

This work was supported by Associazione Italiana per la Ricerca sul Cancro (AIRC) through the Investigator Grant n°23719 and 5x1000 Grant n°12182, by the Italian Ministry of Health through the Ricerca Finalizzata 2016 GR-2016-02361134 grant and by the patient associations “Nastro Viola” and “Voglio il Massimo” through their donations to DaM.

## Conflict of Interest

The authors declare that the research was conducted in the absence of any commercial or financial relationships that could be construed as a potential conflict of interest.
